# Harnessing Proteogenomics to Advance Precision Oncology: From Melanoma and Hepatocellular Carcinoma Perspective

**DOI:** 10.1007/s11912-026-01764-9

**Published:** 2026-04-11

**Authors:** Abolaji Samson Olagunju, Ayoade Desmond Babalola, Sarah Eseroghene Najophe, Oluwabukola Mary Farodoye, Titilayomi Ayomide Otenaike, Umin-Awaji Sunday Godswill, John Oluwafemi Teibo

**Affiliations:** 1 Institute of Biomedical Sciences, University of São Paulo, São Paulo, Brazil; 2 IRCCS, Humanitas Research Hospital, Milan, Italy; 3 Graduate Program in Genetics and Molecular Biology, Federal University of Rio Grande do Sul (UFRGS), Porto Alegre, RS Brazil; 4 Graduate Program in Biochemistry, Federal University of Rio Grande do Sul (UFRGS), Porto Alegre-RS, Brazil; 5 Department of Biochemistry and Immunology, Ribeirão Preto Medical School, University of São Paulo, Ribeirão Preto-São Paulo, Brazil; 6 Department of Medical Imaging, Hematology and Oncology, Ribeirao Preto Medical School, University of São Paulo, Ribeirao Preto-São Paulo, Brazil

**Keywords:** Proteogenomics, Precision oncology, Molecular profiling, Multi-omics, Cancer management, Personalized therapy

## Abstract

**Purpose of Review:**

Genomic profiling has significantly advanced precision oncology; however, relying solely on genome-driven approaches is often insufficient to capture the full molecular complexity of cancer. Proteogenomics integrates proteomics, transcriptomics, and genomic analyses to provide a more holistic view of tumor biology by revealing how genetic alterations translate into functional consequences at the protein level.

**Recent Findings:**

This multidimensional framework enhances the ability to identify clinically actionable biomarkers, uncover dysregulated pathways, and understand tumor heterogeneity with greater precision. By advancing tumor molecular profiling, proteogenomics offers the potential to refine tumor classification, improve diagnostic accuracy, and better tailor therapeutic strategies to individual patient needs. This review examines the expanding role of proteogenomics as an essential tool in personalized cancer management. Key analytical platforms, including mass spectrometry–based proteomics and phosphoproteomics, next-generation sequencing, and integrative computational pipelines, are discussed.

**Summary:**

We also highlight illustrative applications across diverse malignancies, including melanoma and hepatocarcinoma (HCC), where proteogenomic insights have informed therapeutic decision-making, revealed novel drug targets, and improved understanding of treatment resistance mechanisms; challenges and future prospects were also discussed. These advancements collectively highlight the increasing significance of proteogenomics in the evolution of precision oncology and the centrality of integrated molecular profiling in personalized cancer treatment.

**Graphical Abstract:**

**Figure:**
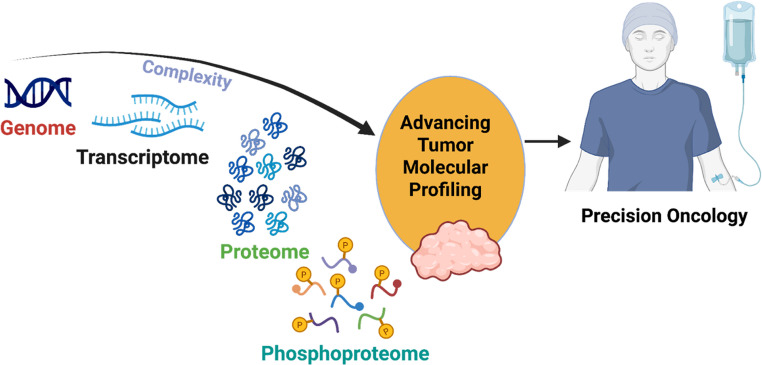
The figure illustrates the integrative use of multi-omics approaches, including genomics, transcriptomics, proteomics, and phosphoproteomics, in advancing cancer profiling and guiding the design of personalized treatment strategies. Genomic analysis provides insights into DNA mutations and structural variations, while transcriptomic profiling captures gene expression patterns that reflect tumor biology. Proteomic studies reveal the abundance and functional state of proteins, and phosphoproteomic data highlight dynamic signaling pathways through post‑translational modifications. Together, these complementary layers of information enable a comprehensive characterization of tumor heterogeneity, the identification of actionable biomarkers, the elucidation of mechanisms for overcoming drug resistance, and the development of tailored therapeutic interventions aimed at improving patient outcomes (created on biorender.com)

## Key Points

Proteogenomics is an integrative multi-omics paradigm that combines genomics, transcriptomics, and mass spectrometry–based proteomics to provide a holistic view of tumor heterogeneity, capturing functional consequences at the protein level that genomics alone often misses.

Genomics, utilizing technologies like next-generation sequencing (NGS) and CRISPR-based tools, has been instrumental in cataloging somatic mutations and developing targeted therapies by providing a comprehensive genomic profile for cancer patients.

Transcriptomics, through methods such as bulk RNA-Seq, single-cell RNA sequencing (scRNA-seq), and spatial transcriptomics, provides functional annotation of the genome, offering dynamic patterns of gene expression and insights into the cellular heterogeneity and spatial context of tumors.

Proteomics and phosphoproteomics, relying on mass spectrometry, are vital for directly measuring protein expression, post-translational modifications, and protein network rewiring, which are the principal effectors of cellular function and the targets of most anticancer therapies.

The approach is essential for advancing precision oncology by refining tumor classification, identifying clinically actionable biomarkers, and better tailoring therapeutic strategies to individual patient needs, focusing on melanoma and hepatocellular carcinoma (HCC).

## Introduction

Decades of genomic research have fundamentally transformed cancer biology, enabling systematic cataloguing of somatic mutations and the development of targeted therapies [1–3]. Yet, despite these advances, the complexity of certain diseases and tumor behavior frequently eludes prediction from DNA sequence alone [2, 4]. Genomic alterations do not always translate into functional consequences at the protein level, nor do they fully capture dynamic regulatory processes such as alternative splicing, post-translational modification, or protein degradation [5]. This discordance limits the clinical utility of genomics alone in precision oncology, where an accurate understanding of tumor phenotype is essential for effective diagnosis, prognostication, and therapeutic decision-making [6, 7].

**Fig. 1:**
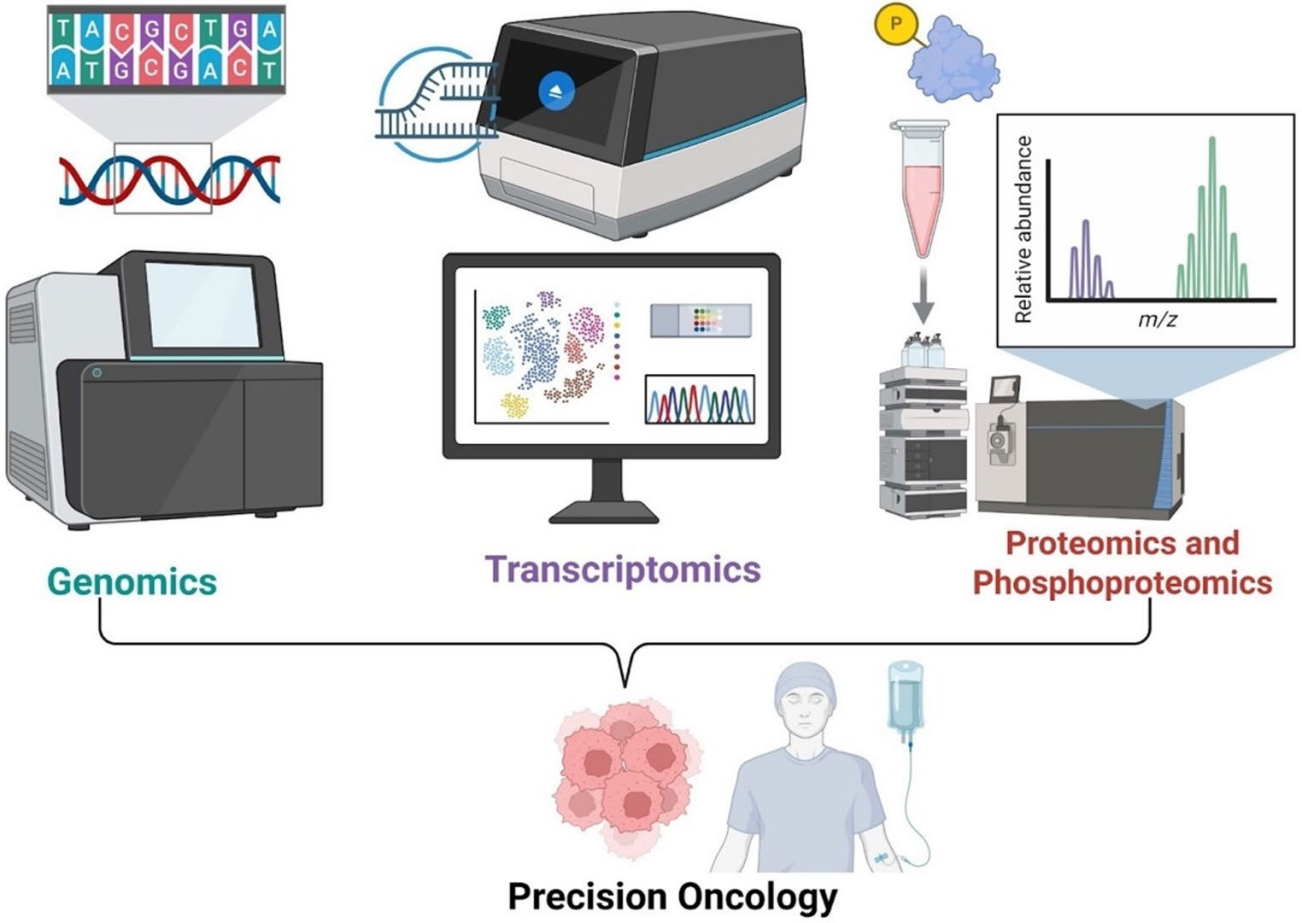
Various omics technologies used in proteogenomics. Comprehensive multi-omics approaches: including genomics, transcriptomics, proteomics, and phosphoproteomics provide an integrated framework for identifying molecular alterations that drive cancer progression and treatment vulnerability, ultimately providing a robust precision understanding on patient tumour and designing precision therapeutics (created on biorender.com)

Proteogenomics has emerged as an integrative multi-omics paradigm that directly addresses this translational gap. By combining liquid chromatography mass spectrometry–based proteomics with patient-specific genomic and transcriptomic data, proteogenomics enables comprehensive mapping of the proteome in its genetically informed context [5] (Fig. 1). Unlike conventional proteomic analyses that rely on canonical protein databases [8, 9], proteogenomic workflows leverage personalized sequence information to detect variant peptides, novel splice isoforms, and tumor-specific antigens, thereby revealing proteomic diversity that would otherwise remain hidden [5]. This approach acknowledges a fundamental biological truth that proteins are the principal effectors of cellular function and the principal targets of most anticancer therapies [10].

The value of proteogenomics in cancer research is now being realized through large-scale efforts that integrate multi-layered data to refine tumor classification, uncover biomarkers of response and resistance, and identify actionable therapeutic targets [11]. For example, proteogenomic analyses have revealed that post-translational modifications and protein network rewiring can drive oncogenic phenotypes independent of genomic alterations, underscoring the need for functional proteomic information in precision oncology [12]. Moreover, proteogenomics can validate the expression of putative neoantigens and guide immunotherapy strategies by directly confirming peptide presentation [6, 13]. The clinical and biological value of proteogenomics is particularly well illustrated in skin cancer (melanoma) and liver cancer (hepatocellular carcinoma), two malignancies characterized by pronounced molecular heterogeneity and variable therapeutic response.

Melanoma, despite being one of the most genomically characterized cancers, exhibits substantial phenotypic diversity that is poorly predicted by mutational status alone [14, 15]. Proteogenomic analyses have enabled refined stratification of metastatic melanoma by integrating protein expression, post-translational modification patterns, and signalling network activity with genomic alterations, thereby corroborating histopathological subtypes and uncovering functional states linked to invasion, immune evasion, and treatment resistance [14–16]. These studies highlight how protein-level regulation and pathway rewiring, rather than genomic mutations per se, can dominate disease behavior and influence clinical outcomes.

Liver cancer, predominantly hepatocellular carcinoma (HCC), represents a complementary model in which etiological diversity and metabolic dysregulation intersect with oncogenic signalling activation [17, 18]. Large-scale proteogenomic studies have revealed distinct molecular subclasses of HCC defined by metabolic reprogramming, kinase activation, and dysregulated cellular processes that are not readily inferred from genomic data alone [19]. In hepatitis B virus–associated HCC, integrated proteogenomic analyses have identified prognostic biomarkers and virus–host interaction signatures with potential translational relevance, offering insights into tumor biology that may inform patient stratification and therapeutic targeting [20]. Melanoma and HCC exemplify how proteogenomics transcends descriptive genomics to deliver mechanistic and clinically actionable insights, reinforcing its emerging role as a foundational approach in precision oncology.

This review provides an integrated overview of recent advances in proteogenomic technologies and the analytical methodologies that support them, highlighting how these innovations are reshaping our understanding of cancer biology, refining molecular classification, and reinforcing the foundations of precision oncology. We examine the growing influence of proteogenomic approaches on clinical cancer management, particularly in biomarker discovery and therapeutic stratification. We also address key challenges, including the complexity of multi-omic data integration, issues of reproducibility, and the need for robust interpretive frameworks capable of translating molecular insights into actionable clinical strategies. Finally, we outline future directions, emphasizing how continued progress in proteogenomics may accelerate the transition toward truly personalized cancer care, in which treatment decisions are informed by a comprehensive, multi-layered understanding of individual tumor biology.

## Precision Oncology

The heterogeneity of cancer remains noteworthy [21]. Tumors can display marked variations in their genetic, epigenetic, phenotypic, and transcriptomic makeup, originating from the same tissue (intratumoral heterogeneity), a single patient but different metastatic sites (spatial heterogeneity), or evolving over time (temporal heterogeneity) [22, 23]. In tandem, individuals with cancers originating from the same tissues or organs can have distinct clinical phenotypes, molecular signatures, disease progression patterns, and responses to treatments [21]. This biological heterogeneity has exposed the impracticability of using conventional one-size-fits-all approaches in diagnosing, treating, and managing patients and tumors, and ultimately achieving sustainable and positive health outcomes [24, 25]. Consequently, there has been a shift in attention towards approaches that prioritize the individuality of patients and tumor types; against this backdrop, precision oncology has gained prominence [26, 27].

Precision oncology entails the identification of molecular alterations unique to patients and the type of cancer affecting them, and the use of therapies to specifically target such alterations [26]. It can also be described as the delivery of the right treatment at the right dose and time to the right patient [25, 28]. This focus on the patient, unlike with the generalized focus of traditional oncology, is so as to produce more effective clinical outcomes, reduce treatment safety concerns, and enhance patients’ life quality [29]. Precision oncology, in addition to targeted diagnosis and treatment, is also employed to improve prognosis, risk assessment, patient follow-up, and the accurate prediction of resistance to therapy among cancer patients [30].

Its origin can be traced back to the late 1990 s, when imatinib was identified as effective against chronic myeloid leukemia harboring the BCR-ABL fusion protein [31]. With this milestone and the advent of technologies like fluorescence in situ hybridization (FISH) and polymerase chain reaction (PCR), alterations unique to specific cancers (e.g., NSCLC, melanoma, and pancreatic cancer), including mutant forms of EGFR, BRAF, and RAS, among other genes, were identified [32]. This then facilitated the use of targeted treatments, including tyrosine kinase inhibitors - erlotinib and gefitinib; programmed cell death protein 1 (PD-1) inhibitor—pembrolizumab; and neurotrophic tyrosine receptor kinase (NTRK) inhibitor, for example, entrectinib, among others [33–36].

Over the years, precision oncology has gained increasing traction, particularly with the emergence of advanced technologies such as genomics, proteomics, and transcriptomics which provide critical insights into patient/tumor characteristics, enabling effective therapeutic decision-making [37, 38]. For instance, the genome profiling of melanoma and HCC samples using next-generation sequencing (NGS) technologies has revealed unique aberrations – single-nucleotide variations, copy-number alterations, and structural variations that drive carcinogenesis [39–42]. NGS, when used to sequence melanoma patients’ samples, has also revealed resistance-associated mutations (*MITF*^R316K^, *CDK4* ^R24^, *PTEN*, *CTNNB1*^S45F^) and allowed for the robust categorization of hepatocellular carcinoma (HCC) [43–45]. All of these have enhanced diagnosis, accurate patient matching to clinical trials based on their disease state and tumor mutations, and ultimately improved treatment responses and patient survival [46, 47].

Proteomics technologies such as mass spectrometry and reverse-phase protein array (RPPA) have also been used to detect epigenetic changes, quantify protein biomarkers, identify metabolite patterns within tumor microenvironments, and improve drug dosage measurement [25, 48]. Across cancers, including liver, pancreatic, bladder, and breast, these have boosted the efficacy of clinical screening and ensured proactive approaches towards treatment resistance (detection of drug resistance signatures and integration of alternative therapies) and reduced drug exposure toxicity [49–51].

Despite these advances, the limitations of single omics technologies constrain precision oncology and underscore the need for integrated approaches. Emerging suggestions emphasize the use of integrated/multi-omics approaches, such as proteogenomics, because they can thoroughly capture the complexity of tumors and patients’ molecular profiles, thereby unlocking the optimum potential of personalized cancer care.

## Different Omics Technologies

- **Genomics**.
  Genomics is a data-driven science focused on the comprehensive study of genomes and genomic sequences of organisms through various technological approaches. Early approaches, such as Sanger sequencing, PCR-based technologies, and FISH, laid the foundation for genome analysis, while more recent approaches, such as next-generation sequencing (NGS), have revolutionized genome profiling and enabled precise genetic profiling. NGS is a DNA sequencing technology that enables the sequencing of the whole genome and the whole exome, as well as transcriptomic, chromatin, and epigenetic profiling data [52]. Advances in these assay technologies have facilitated an evolution from single-gene testing toward comprehensive genomic profiling. By sequencing the whole genome, there is a better understanding of genetic variation across species, cells, tissues, and disease states. In addition, NGS enables identification of diverse genomic alterations, including point mutations, gene fusions, structural rearrangements, and clinically relevant biomarkers. As a result, genomics has been promising in elucidating disease mechanisms and improving prevention, diagnosis, and treatment strategies. Beyond the sequencing of the genome, genomics also enables the exploration and manipulation of the genome to experimentally define the mechanisms underlying gene function and regulation. Clustered Repeat Interspaced Short Palindromic Repeats (CRISPR)-based functional genomic tools have been instrumental, due to their scalability and multiplex ability in gene editing and exploration [53].
  These genomic editing technologies enable direct connections between genotype and phenotype, determine the particular effect of gene products, and allow the identification of genes involved in specific cellular processes. The advent of these genomic technologies has been particularly impactful in the era of cancer precision medicine, where a comprehensive genomic profile is generated for cancer patients and used for treatment decisions. Large-scale genomic characterization studies have shown that cancer initiation and progression are partly driven by genomic alterations and that the genomic drivers can differ substantially between patients [54]. These have been instrumental in the conception of initiatives such as The Cancer Genome Atlas (TCGA) and the International Cancer Genome Consortium (ICGC). Together with the Human Genome Project, these projects have revealed information on how genetic variation in both coding and non-coding parts of the genome contributes to the development of cancer. Furthermore, NGS-based approaches have enabled the detection of complex genomic abnormalities associated with tumor growth and evolution, including chromosomal rearrangements, intra-tumor heterogeneity, and immunogenomic features [55] (Fig. 1). The CRISPR genome-editing tools also allow precise gene modifications to systematically identify essential cancer-causing and cancer-preventing genes. It is also used to modify the tumor microenvironment and identify gene resistance mechanisms. Beyond therapeutic applications, CRISPR-based platforms are also used for cancer diagnostics and early cancer detection [56].
- **Transcriptomics**
  Transcriptomics is the comprehensive analysis of the complete set of RNA transcripts (the transcriptome) produced by the genome using high-throughput methods. By enabling the cataloging of all types of transcripts, including mRNA, tRNA, lncRNA, siRNA, miRNA etc., transcriptomics provides functional annotation of the genome and an insight into the dynamic pattern of gene expression under different physiological and pathological conditions. As a result, transcriptomics is being broadly applied across biological, clinical, and pharmaceutical research for disease diagnosis, prognosis, and biomarker discovery [57].
  Initially, transcriptome profiling was characterized using microarray technology and was later extended to sequencing upon the advent of next-generation sequencing. Sequencing-based transcriptomics offers a broader dynamic range than microarrays due to its increased sensitivity and capacity to detect novel transcripts and biologically critical isoforms, making it the most widely used transcriptomic approach to date [58]. Recent technological advances have seen the evolution of sequencing transcriptomics from traditional bulk levels to single-cell and spatial location levels. Traditional bulk RNA-Seq involves sequencing the whole transcriptome library, and it remains a standard method for transcriptome analysis. Depending on the goal of the experiment, bulk RNA-Seq may use single-end short-read sequencing, commonly used for differential gene expression, or paired-end long-read sequencing, which enables the detection of alternative splicing, point mutations, and gene fusions in addition to the analysis of differentially expressed genes. Single-end short-read sequencing is primarily focused on mRNA, making it a simple, cost-effective, and widely used approach [59].
  The bulk approach of the traditional RNA-seq enables the analysis of thousands of cells, which masks the nuances of individual cell gene expression, resulting in the loss of cell type-specific understanding and spatial context of gene expression [60]. The single-cell RNA sequencing (scRNA-seq) and spatial transcriptomic approaches are recent advances that address these limitations. scRNA-seq enables transcript sequencing at the level of individual cells, providing a comprehensive insight into the functional heterogeneity of tissue samples and cell populations. This approach has been instrumental in constructing a reference catalogue of gene expression in individual cells, allowing the identification and functional characterization of cell types and subtypes [61]. However, scRNA-seq requires tissue dissociation, which results in loss of spatial context.
  Spatial transcriptomics overcomes this limitation by providing whole transcriptome data with spatial information. It also facilitates the decoding of cellular communication networks within the tissue microenvironments, enabling the tracking of dynamic changes that occur during development and disease progression [62].
  To date, transcriptomics technology has been extensively applied in precision oncology, where it has enabled the comprehensive profiling of cancer cells and their surrounding microenvironments. These approaches have facilitated the discovery and validation of novel malignant cell populations, revealed the heterogeneity of tumor niches, and supported the generation of detailed molecular and cellular atlases of cancer disease. These advances have enabled increased accessibility to comprehensive genomic profiling and driven rapid development of targeted therapies and precision drug discovery strategies.
- **Proteomics and Phosphoproteomics**
  Proteomics is an integral part of proteogenomics, which involves various techniques for analyzing the proteome, facilitating the identification, quantification, and characterization of proteins and their post-translational modifications in a variety of biological samples, such as cells, tissues, and biofluids [63]. Proteomics offers crucial insights into the biochemical processes that govern cellular expression, localization, and signaling pathways by capturing proteins that are actively modified and functional [64, 65]. Recent developments in mass spectrometry–based proteomics have enhanced the precision and consistency of protein quantification, enabling the exploration of biological changes associated with disease, signaling pathways, and drug mechanisms. High-throughput liquid chromatography coupled with tandem mass spectrometry (LC–MS/MS) serves as a pivotal platform in proteomics, generating high-resolution datasets that facilitate comprehensive structural and functional characterization of proteins and protein complexes [65, 66] (Fig. 1).
  Four central mass spectrometry-based proteomic acquisition strategies are currently utilized: two untargeted methods, data-dependent acquisition (DDA) and data-independent acquisition (DIA), and two targeted methods, selective/multiple reaction monitoring (SRM/MRM) and parallel reaction monitoring (PRM) [67]. These strategies are commonly employed in clinical and translational research to quantify protein abundance under various conditions, using either label-free or isotope-labeled workflows [68, 69]. Targeted proteomics emphasizes the accurate quantification of specific protein panels via proteotypic peptides, attaining improved sensitivity and reproducibility through selective ion monitoring [63, 64, 70].
  Data-dependent analysis facilitates extensive proteome analysis by targeting the most prevalent precursor ions for fragmentation and identification, commonly employing high-resolution accurate mass (HRAM) analyzers like Orbitrap or time-of-flight (TOF) instruments. However, the stochastic nature of precursor selection may limit reproducibility and lead to under-sampling [71]. DIA mitigates these limitations by fragmenting all precursor ions within specified mass-to-charge (m/z) windows, thus integrating the coverage of DDA with the reproducibility of targeted methods [71, 72]. DIA produces intricate MS2 spectra as a result of peptide co-fragmentation; however, recent developments in bioinformatics and spectral library–free methodologies facilitate direct and effective analysis of DIA data [73, 74].
  Quantitative proteomics encompasses various chemical and metabolic labeling strategies, including Tandem Mass Tag (TMT), Isotope Coded Affinity Tag (ICAT), Isobaric Tags for Relative and Absolute Quantification (iTRAQ), and Stable Isotope Labelling by Amino Acids in Cell Culture (SILAC) [75]. Recent technological innovations, such as Field Asymmetric Ion Mobility Spectrometry (FAIMS), Synchronous Precursor Selection-Ms/MS/MS (SPS-MS3), and real-time data acquisition, have improved the depth of proteome analysis and the accuracy of quantitative measurements. Label-free DIA has become increasingly significant in proteogenomic and multi-omics research owing to its scalability and lower rates of missing data [11].
  Next-generation instruments such as the Orbitrap Astral and timsTOF platforms provide exceptional speed, sensitivity, and resolution, facilitating high-throughput bulk and single-cell proteomics [76–78]. These advances enhance the clinical and biological applications of proteomics, facilitating biomarker discovery, therapeutic target identification, and insights into disease progression [79–81].
  To increase the complexity of proteomics data and identify specific sites on proteins responsible for regulating protein function in cancer, MS-based phosphoproteomics has significant potential for identifying activated signalling networks and new therapeutic targets in cancer cells [82]. Unbiased techniques have demonstrated the comprehensive scope of the phosphoproteome and provided insights into the dynamics of insulin signaling [83]. Phosphoproteomics encompasses several key steps: (i) Solubilization of proteins from a sample; (ii) removal of contaminants, including detergents and lipids, often achieved through protein precipitation; (iii) proteolytic digestion, typically employing trypsin and Lys-C; (iv) enrichment of phosphopeptides, commonly utilizing titanium dioxide or antibodies; (v) peptide desalting; (vi) liquid chromatography tandem mass spectrometry (LC-MS/MS); and (vii) database searching and data analysis [83]. The output generally includes a list of phosphopeptides, specifying the phosphorylation sites within the peptides and their abundance, as assessed by the quantification methods utilized in the study, including label-free quantification, metabolic labeling, or isobaric tags [84]. These proteomics tools are essential to proteogenomics, which drives precision oncology through the identification of protein targets, the understanding of the mechanisms of action of molecular effectors, the unravelling of signalling pathways, and the elucidation of drug resistance mechanisms. As technology evolves, plasma proteomics, single-cell, and spatial proteomics will help enhance proteomics applications in clinical settings since proteomics captures fine details of the cellular workforce (proteins).

### Technologies and Methodological Approaches in Proteogenomics

Technological advancements over the past two decades have driven the emergence of proteogenomics, a multidisciplinary field that integrates genomic, transcriptomic, and proteomic data to achieve a more comprehensive understanding of gene expression and protein function [85]. Proteogenomics originated from early efforts to improve genome annotation by leveraging proteomic evidence, most notably through proteogenomic mapping approaches that used mass spectrometry (MS) data to validate and refine predicted genes, as demonstrated in *Mycoplasma pneumoniae* and other model systems [86, 87]. Since then, the field has evolved rapidly alongside advances in high-throughput sequencing, mass spectrometry, and computational biology.

**Fig. 2:**
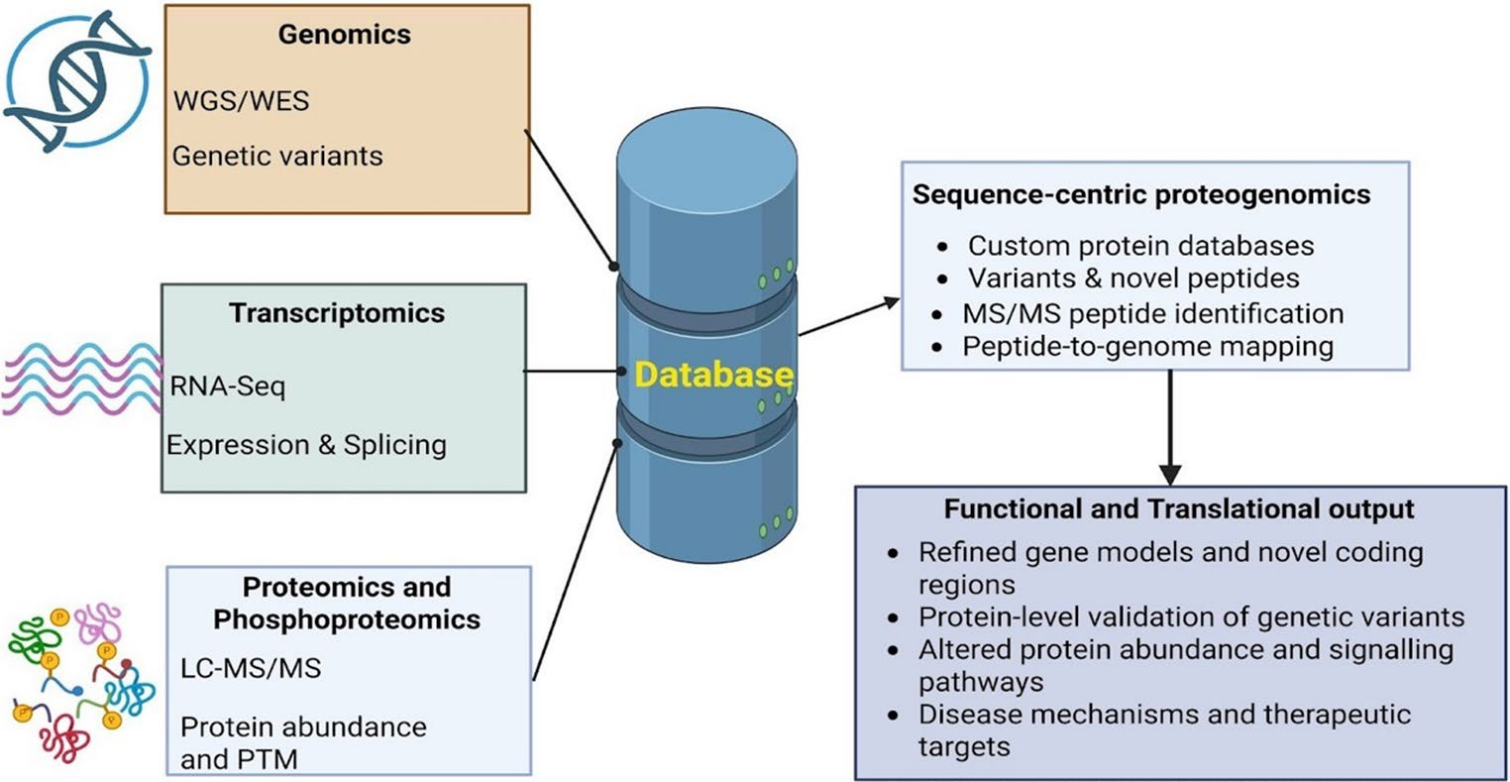
Integrative and analytical workflow domains in proteogenomics. Proteogenomics integrates genomic, transcriptomic, and proteomic data to link genetic variation with functional protein products. Genomic and transcriptomic sequencing data are used to construct custom protein sequence databases incorporating sequence variants, alternative splice junctions, and novel open reading frames. Tandem mass spectrometry–based proteomics enables peptide identification through database searching, followed by mapping of peptides to genomic coordinates to refine gene models and validate protein-level consequences of genomic alterations. WGS- Whole Genome Sequencing, WES- Whole Exon Sequencing, RNA-Seq—RNA sequencing, LC-MS/MS—liquid chromatography-mass spectrometry, PTM - Posttranslational modification (created on biorender.com)

At its core, proteogenomics combines DNA sequencing–based genomics, RNA sequencing–based transcriptomics, and MS-based proteomics [5, 88]. Genomics provides the complete DNA sequence and identifies genetic variants, including single-nucleotide variants, insertions, deletions, and structural rearrangements [87]. Transcriptomics, primarily through RNA sequencing (RNA-seq), offers insights into gene expression levels, alternative splicing, and transcript isoform diversity. Proteomics, in turn, directly measures protein expression, post-translational modifications, and protein abundance, thereby capturing biological information that cannot be fully inferred from nucleic acid data alone. The integration of these complementary layers enables the direct linking of genomic variation to functional protein products (Fig. 2).

Proteogenomic methodologies have been broadly categorized by Ruggles et al. [87] into four interrelated domains: sequence-centric proteogenomics, analysis of proteogenomic relationships, integrative modelling of multi-omics data, and data sharing and visualization. Among these, sequence-centric proteogenomics represents the foundational approach and remains central to many applications. This strategy focuses on integrating genomic and proteomic data to refine gene models, discover novel coding regions, and detect protein-level consequences of genomic variation, including single amino acid variants, alternative splice junctions, insertions, deletions, and gene fusions.

Central to sequence-centric proteogenomics is tandem mass spectrometry (MS/MS), which enables peptide identification and protein inference [85]. In a typical bottom-up proteomics workflow, proteins are enzymatically digested into peptides, separated by liquid chromatography, and analyzed by MS/MS [5, 89].The resulting spectra are interpreted using database search algorithms that compare experimental spectra against theoretical spectra derived from peptide sequences in a reference database [89, 90]. In proteogenomics, these databases are often custom-constructed to include variant-specific sequences derived from whole-genome sequencing (WGS), whole-exome sequencing (WES), or RNA-seq data. To facilitate novel gene discovery, peptide spectra may also be searched against six-frame translations of the reference genome, allowing detection of previously unannotated open reading frames [87].

Proteomics-assisted genome annotation has proven particularly valuable in refining gene models and validating predicted coding regions. Peptides identified through proteogenomic searches are mapped back to genomic coordinates, providing direct experimental evidence of translation. This evidence has been used to confirm exon–intron boundaries, correct start sites, identify alternative reading frames, and train computational algorithms for improved gene prediction [91]. Such approaches have been especially impactful in non-model organisms and poorly annotated genomes, where transcriptomic or computational predictions alone may be insufficient.

Beyond gene annotation, advances in quantitative proteomics have expanded the scope of proteogenomics. Techniques such as isobaric labeling (e.g. TMT) enable multiplexed quantification of proteins across multiple samples, while data-independent acquisition (DIA) approaches, including Sequential Window Acquisition of all Theoretical Fragment Ion Spectra (SWATH-MS), provide increased proteome coverage and reproducibility [92, 93]. These technologies facilitate the systematic analysis of how genomic alterations influence protein abundance and signaling pathways across biological conditions.

Computational integration represents another critical technological pillar of modern proteogenomics. Statistical and machine learning–based methods are increasingly employed to model relationships between genomic variants, transcript abundance, and protein expression. These integrative approaches are essential for deciphering complex regulatory mechanisms, particularly in disease contexts such as cancer, where proteogenomics has been instrumental in linking somatic mutations to dysregulated signalling networks and therapeutic vulnerabilities [94, 95].

Finally, effective data visualization, standardization, and sharing remain ongoing challenges due to the scale and complexity of proteogenomic datasets. Genome browsers, network visualization tools, and community resources have been developed to support data interpretation and dissemination. Large-scale initiatives have further accelerated technological development by establishing standardized workflows and publicly accessible datasets, thereby promoting reproducibility and cross-study comparisons [86] (Fig. 2).

Collectively, continued innovations in sequencing technologies, mass spectrometry, and computational analysis have transformed proteogenomics into a powerful framework for functional genome interpretation. As these technologies mature, proteogenomics is increasingly positioned as a critical approach for elucidating the molecular consequences of genetic variation in both basic and translational research.

## Proteogenomics as a Translational Tool for Precision Target Discovery

An effective proteogenomic approach starts with a specific biological and clinical question, such as mechanisms of resistance, identifying variables influencing therapeutic response, and (bio) markers of invasive disease. The study methodology must delineate the comparison categoris for example, primary versus metastatic tumors, responders vs. non-responders, or hot versus cold phenotypes, since these differences impact all subsequent analyses. Samples from clinical trials, presumably Phase III, will be important in generating primary evidence for modifications in clinical practice centered on proteogenomic principles. This necessitates a commitment to gather appropriate specimens, including meticulously curated and annotated clinical data, to achieve significant translational targets [11].

Satpathy et al. [96] demonstrated a significant advancement in proteogenomics in the implementation of a microscaled TMT-based discovery method utilizing minimal protein quantities extracted from optimal culturing temperature (OCT)-embedded core needle biopsies, facilitating the acquisition of the proteome, including post-translational modifications (PTMs), from the same cores as nucleic acids (DNA and RNA). This is a crucial subsequent step, as in cases with locally advanced or metastatic cancer, systemic treatment generally commences after diagnosis rather than surgery, indicating that a core needle biopsy is the sole chance to acquire tumor tissue in its unaltered condition. Upon completion of a discovery experiment, both supervised and unsupervised proteogenomic studies are performed in relation to the primary endpoint of the study, usually the therapeutic response, leading to the formulation of biological and clinical hypotheses. The validation of these ideas might require the application of targeted, MS-based assays or alternative multiplexed immunoassays. This strategy will be applicable to all cancer types and stages.

Furthermore, after early studies on colon, ovarian, and breast cancer [97–99], several other assessments using an array of proteogenomic landscape research spanning numerous types of cancer have been released [100, 101]. These studies carefully examined and integrated genomic, transcriptomic, proteomic, and post-translational modification (PTM) data to enhance biological understanding of disease pathophysiology and uncover therapeutic targets for each cancer type [11] showing that proteomics and phosphoproteomics are transforming cancer research by uncovering functional protein signaling pathways that genomics fails to identify, thus offering novel treatment targets and biomarkers for targeted oncology.

Melanoma, an aggressive form of skin cancer representing 75% of deaths from this type of cancer [102], has been thoroughly investigated at the genetic level; nevertheless, proteomics offers a more immediate assessment of cellular function. Mass spectrometry-based proteomics facilitates comprehensive analysis of protein abundance, isoforms, and post-translational changes, uncovering tumor-specific proteins that promote progression and resistance. Research indicates that melanoma cells exhibit unique proteome profiles, such as inosine-5’-monophosphate dehydrogenase 2, galectin-1, serine/threonine-protein phosphatase, cyclophilin A, protein DJ-1, and cofilin-1 [103], in contrast to normal melanocytes, characterized by the overexpression of immune evasion proteins, metabolic enzymes, and stress response factors. These protein-level insights reveal druggable limitations that may not be obvious from transcriptome data [104, 105]. In addition, phosphoproteomics enhances understanding by delineating phosphorylation events, which are essential regulators of kinase signaling. The progression of melanoma and resistance to therapy frequently entails reconfigured kinase networks, including the PI3K/AKT and MAPK pathways. Phosphoproteomic profiling has revealed hyperactive substrates and kinases that may serve as possible therapeutic targets, notably receptor tyrosine kinases as well as downstream effectors. This effectively captures dynamic signaling alterations in response to targeted therapies, elucidating bypass pathways and feedback loops that foster resistance [104].

Xiang et al. (2024) [106] performed a multivariate study indicating that PRKDC gene amplification serves as a predictive biomarker for melanomas. Subsequent proteogenomic analysis, together with functional tests, indicates that the cis-effect of PRKDC amplification may promote tumor proliferation by activating DNA repair and folate metabolism pathways. The proteome-based category of primary melanomas distinguishes three prognosis-associated subtypes: the extracellular matrix (ECM), cell proliferation, and the angiogenesis subtype (characterized by a high metastasis rate). This classification establishes a critical framework for the application of targeted therapies tailored to specific melanoma subtypes. Furthermore, an independent anti-PD-1 treatment cohort indicates that the activation of the MAPK7-NFKB signaling pathway may enhance T-cell recruitment and augment patient sensitivity to immunotherapy.

Proteomics, on the other hand, is essential for elucidating the mechanisms of clinical pharmacotherapy, especially in the context of personalized therapy and drug resistance studies. Ongoing technological improvements are anticipated to enhance the comprehensiveness and detail of proteomics applications in the analysis of therapeutic pharmacological mechanisms [107]. Emerging spatial proteomics and phosphoproteomics findings highlight the significance of the tumor microenvironment (TME) in melanoma. Proteomic mapping of melanoma during T-cell assault has uncovered protein-level modifications that facilitate immune evasion, including changes in antigen presentation and expression of the checkpoint ligand [108]. These results indicate novel treatment possibilities encompassing a focus on immunomodulatory proteins and phospho-signaling nodes that modulate T cell infiltration and functionality.

Incorporating proteomics into immunotherapy studies enables scientists to discover biomarkers that predict responses and develop appropriate combination approaches; for example, trametinib in combination with dabrafenib [109], ceritinib [110], mebendazole [111] has been shown to produce an effective response against melanoma. Similarly, tremelimumab [112] and ipilimumab [113] are CTLA-4 inhibitors approved by the FDA for metastatic melanoma. Several clinical studies employed various biomarkers, drugs, or pathway-specific inhibitors across different types and stages of melanoma **(**Table 1**)**. These clinical studies encompass phases I to III and collectively demonstrate the evaluation of immune checkpoint blockade, targeted treatments, and combination regimens in both resectable and unresectable melanoma. Alongside evaluating clinical efficacy and safety, numerous studies integrate exploratory and mechanistic biomarkers such as PD-L1 expression, BRAF mutation status, circulating tumor DNA, pathological response rates, and immune cell signatures to enhance patient stratification and elucidate the factors influencing treatment response and resistance [14]. Collectively, these trials demonstrate the advancing terrain of melanoma treatment, whereby strategic medication combinations and biomarker-based methodologies are merging to enhance therapeutic outcomes and guide forthcoming precision immuno-oncology initiatives.

**Table 1:** Selected phase I–III melanoma clinical trials

Trial (common name)	Phase	Setting/patients	Intervention type	Biomarkers/Drugs	Main objective	References
KEYNOTE-716	III	Resected stage IIB/IIC cutaneous melanoma	Pembrolizumab vs. placebo (adjuvant)	PD-1	Improve recurrence-free survival in high-risk stage II melanoma	[120]
KEYNOTE-006	III	Unresectable/metastatic melanoma	Pembrolizumab vs. ipilimumab	PD-1 vs. CTLA-4; PD-L1, LDH, BRAF	Demonstrate OS and PFS superiority of pembrolizumab vs. ipilimumab	[121]
CheckMate 067	III	Advanced melanoma, treatment-naïve	Nivolumab, ipilimumab, or combo	Nivolumab+ipilimumab; PD-L1 and BRAF as stratifiers	Test whether nivolumab±ipilimumab improves OS vs. ipilimumab monotherapy	[122]
CheckMate 238	III	Resected stage IIIB–C/IV melanoma	Adjuvant nivolumab vs. ipilimumab	Adjuvant PD-1 vs. CTLA-4; PD-L1 and stage risk groups	Improve recurrence-free survival with better tolerability than ipilimumab	[123]
RELATIVITY-047	II/III	Unresectable/untreated metastatic melanoma	Nivolumab + relatlimab vs. nivolumab	Dual PD-1 + LAG-3 blockade	Determine if LAG-3 combination improves PFS vs. PD-1 alone	[124, 125]
DREAMseq (EA6134)	III	BRAF-mutant metastatic melanoma	Ipi+nivo vs. dabrafenib+trametinib sequencing	Immunotherapy-first vs. targeted-first sequence	Establish optimal treatment sequence for BRAF-mutant melanoma	[126]
COMBI-d	III	BRAF V600E/K metastatic melanoma	Dabrafenib + trametinib vs. dabrafenib	BRAF + MEK dual blockade	Improve PFS/OS vs. BRAF inhibitor monotherapy	[127]
COMBI-v	III	BRAF V600E/K metastatic melanoma	Dabrafenib + trametinib vs. vemurafenib	Comparison of targeted combinations	Show survival advantage of dabrafenib+trametinib vs. vemurafenib alone	[128]
COLUMBUS	III	BRAF-mutant metastatic melanoma	Encorafenib + binimetinib vs. vemurafenib	Alternate BRAF/MEK combo	Improve PFS/OS and toxicity profile with encorafenib+binimetinib	[129]
IMspire150	III	BRAF-mutant metastatic melanoma	Atezolizumab + vemurafenib + cobimetinib vs. vem+cobi	Triplet IO+targeted combination	Test if adding PD-L1 blockade improves PFS beyond doublet targeted therapy	[130]
SWOG S1801	II	Resectable stage III/IV melanoma	Neoadjuvant + adjuvant pembrolizumab vs. adjuvant-only	Neoadjuvant PD-1; pathologic response as key biomarker	Determine if neoadjuvant strategy improves event-free surviva	[131]
OpACIN-neo	II	Stage III nodal melanoma	Neoadjuvant ipilimumab+nivolumab (different dosing cohorts)	Pathologic complete response, T-cell signatures as biomarkers	Optimize dosing of neoadjuvant combo balancing efficacy vs. toxicity	[132]
mRNA-4157 + pembrolizumab	II	Resected high-risk melanoma	Personalized neoantigen mRNA vaccine + PD-1	Neoantigen load, T-cell responses, ctDNA clearance	Reduce recurrence by boosting personalized T-cell responses on top of PD-1	[133]
PD-1 + TIGIT/costimulatory agonists (basket/expansion cohorts	I/II	Resectable melanoma	PD-1 plus TIGIT inhibitor	Immune signature, intratumoral CD8, Fc-effector function	Explore synergy of PD-1 with novel inhibitory or costimulatory pathways	[134]

Furthermore, certain emerging small-molecule targets are being identified and explored using this omics strategy. Despite the lack of sufficient definitive clinical trials, predominantly comprising preclinical studies, *in vitro or in vivo*, the role of small-molecule therapeutics in melanoma treatment should not be neglected. The optimistic findings indicate that future research will concentrate on pharmacological combination therapy or adjuvant therapy. Currently, heat shock protein 90 (HSP90) [114, 115], Bcl-2 [116–118], and ganglioside GD2 [119] are prominent targets in the realm of small molecules.

Hepatocellular carcinoma (HCC) constitutes approximately 75%–85% of all primary liver cancers, characterized by minimal treatment options and a dismal prognosis. The majority of HCC cases are identified at a late stage and can only undergo systemic anti-tumor treatment, which is less potent and exhibits poor response rates. The prognoses of individuals with HCC displayed significant variability, complicating outcome predictions. Therefore, precision staging and therapy are essential for improving clinical outcomes in HCC [135], highlighting the significance of proteomics-based precision typing for this condition.

Xign et al. (2023) [135] investigated multi-omics profiling of primary tumor tissues (T) and corresponding para-tumor tissues (N) from hepatocellular carcinoma (HCC) patients, with subtypes determined using proteomic analysis utilizing DIA. Additionally, an integrated multi-omics study was conducted, focusing on kinase abundance and the kinase-substrate regulation network to investigate the molecular features and identify potentially actionable medications for various HCC proteome subtypes. Three HCC proteome subgroups were found, characterized by metabolism, proliferation, immune response, and metastasis. This comprehensive multi-omics research revealed modifications in mutation patterns, immunological environments, and kinase-substrate regulatory networks across three proteomic subtypes. The drug-targetable proteins identified via proteomic and phosphoproteomic data could provide rationale for broadening treatment options beyond the existing FDA-approved therapies for HCC, which is encouraging for addressing the restricted availability of therapeutic agents and their suboptimal response rates in HCC [19].

Generally in HCC, proteomic technologies are predominantly used for the identification of drug targets, biomarkers, drug action, interaction, and resistance mechanisms, as well as elucidating the pathophysiology of HCC. It is playing an increasingly pivotal role in liver cancer research, enhancing the comprehension of disease causes, screening, detection, and treatment [136, 137]. Additionally, its use is propelling progress in HCC treatment, especially when used in clinical cohort studies. It offers critical perspectives on the molecular mechanisms of HCC, enabling the discovery of early diagnostic markers, aiding the identification of both diagnostic and therapeutic targets, and increasing prognosis evaluation and the development of personalized therapies [135, 138].

Previous studies employed advanced proteomic technologies, including mass spectrometry–based approaches, to comprehensively delineate the proteomic expression profile and phosphoproteomic landscape associated with early-stage hepatocellular carcinoma (HCC). By systematically mapping alterations in protein abundance and phosphorylation signaling networks, these investigations provided critical insights into the molecular mechanisms underlying tumor initiation and progression. Importantly, the integration of proteomic and phosphoproteomic data revealed dysregulated pathways involved in cell cycle regulation, apoptosis, and oncogenic signaling, thereby identifying novel biomarkers and therapeutic targets. Such findings have laid the groundwork for precision medicine strategies in liver cancer, offering opportunities to develop targeted therapies that disrupt aberrant signaling cascades and improve patient outcomes [139, 140]. Several of these targeted therapies have advanced from laboratory development to real-world clinical trials globally; some of these include atezolizumab and bevacizumab [141], sorafenib [142], lenvatinib [143, 144], regorafenib [145], and cabozantinib [146], quinacrine [140], lenvatinib [141–143], osimertinib [141], apatinib [128, 129], and regorafenib [144] are among the targeted therapeutic agents that have received considerable attention recently for their potential in the treatment of HCC. An increasing number of phase I–III clinical trials conducted evaluated these drugs as either monotherapies or in conjunction with established standards of treatment **(**Table 2**)**. These pharmacological drugs inhibit important intracellular signalling pathways in tumor cells, therefore reducing tumor growth, angiogenesis, and metastasis, and are very beneficial in HCC therapy [145]. Although there is a strong correlation between HCC etiology and protein PTMs, studies on protein expression levels and phosphorylation alterations following the administration of certain molecularly targeted therapies remain insufficient.

**Table 2:** Selected phase I–III hepatocellular carcinoma clinical trials

Trial (common name)	Phase	Setting/patients	Intervention type	Biomarkers/Drugs	Main objective	References
SHARP (NCT00105443)	III	Advanced unresectable HCC, mostly Western	Sorafenib vs. placebo	First systemic TKI standard; exploratory analyses of prognostic factors	Demonstrate the OS benefit of sorafenib vs. placebo in advanced HCC	[147]
Asia-Pacific sorafenib trial	III	Advanced HCC, predominantly Asia-Pacific	Sorafenib vs. placebo	Regional validation of sorafenib; viral hepatitis background	Confirm survival benefit and safety of sorafenib in an Asia-Pacific cohort	[148]
REFLECT (lenvatinib vs. sorafenib)	III	First-line unresectable HCC	Lenvatinib vs. sorafenib	Multi-kinase angiogenic TKI; exploratory AFP and tumor burden	Show non-inferiority of lenvatinib to sorafenib for OS	[149]
IMbrave150	III	First-line unresectable HCC	Atezolizumab + bevacizumab vs. sorafenib	IO + anti-VEGF combo; PD-L1, VEGF/angiogenesis markers	Demonstrate superior OS and PFS vs. sorafenib with IO + anti-VEGF	[150]
HIMALAYA (STRIDE regimen)	III	First-line unresectable HCC	Durvalumab + tremelimumab vs. sorafenib	Dual PD-L1 + CTLA-4; immune-related biomarkers	Show OS benefit of STRIDE vs. sorafenib and define IO-only first-line option	[151]
CheckMate 040	I/II	Advanced HCC, sorafenib-naïve and -experienced	Nivolumab monotherapy and nivolumab + ipilimumab cohorts	Dose-finding, safety; PD-L1, etiologic subtype, immune signatures	Define safety/efficacy of PD-1 ± CTLA-4 and support accelerated approval	[152]
CheckMate 459	III	First-line unresectable HCC	Nivolumab vs. sorafenib	IO vs. TKI; PD-L1 and etiology as subgroups	Compare OS and response rates of nivolumab vs. sorafenib	[153]
KEYNOTE-224	II	Sorafenib-pretreated advanced HCC	Pembrolizumab single arm	PD-1 blockade; PD-L1 expression, viral vs. non-viral HCC	Assess ORR and safety of pembrolizumab post-sorafenib	[154]
KEYNOTE-240	III	Advanced HCC after sorafenib	Pembrolizumab vs. placebo	PD-1 s-line; PD-L1 and AFP exploratory	Test OS and PFS improvement vs. placebo in sorafenib-exposed patients	[141]
RATIONALE-301	III	First-line unresectable HCC	Tislelizumab vs. sorafenib	PD-1 vs. TKI; biomarker-rich analyses planned	Demonstrate non-inferiority/superiority in OS and better tolerability	[155]
RESORCE	III	Second-line advanced HCC after sorafenib	Regorafenib vs. placebo	Sequential TKI strategy; AFP and clinical risk factors	Show OS benefit of regorafenib in sorafenib-tolerant progressors	[156]
CELESTIAL	III	Second/third-line advanced HCC	Cabozantinib vs. placebo	Multi-target TKI (MET/VEGFR/AXL); prognostic subgroup analyses	Demonstrate OS and PFS superiority vs. placebo in previously treated HCC	[146]
REACH-2	III	Second-line HCC with high AFP	Ramucirumab vs. placebo	AFP ≥ 400 ng/mL as inclusion biomarker	Validate AFP-high subgroup benefit from VEGFR2 blockade	[157]
ORIENT-32	II/III	First-line unresectable HCC, mainly HBV-related	Sintilimab + bevacizumab biosimilar vs. sorafenib	PD-1 + VEGF inhibition; liver function and etiology subgroups	Demonstrate OS and PFS benefit of IO + anti-VEGF vs. sorafenib	[158]
COSMIC-312	III	First-line unresectable HCC	Atezolizumab + cabozantinib vs. sorafenib	IO + multi-kinase TKI combo; exploratory biomarker work	Assess whether adding cabozantinib to atezolizumab improves PFS/OS vs. sorafenib	[159]
Camrelizumab + rivoceranib (apatinib) trial	III	First-line unresectable HCC	Camrelizumab (PD-1) + rivoceranib vs. sorafenib	PD-1 + VEGFR2 inhibitor; AFP and etiologic subgroup analyses	Compare OS and PFS of IO + TKI vs. sorafenib	[160]
LEAP-002	III	First-line unresectable HCC	Pembrolizumab + lenvatinib vs. lenvatinib	PD-1 + TKI; rich translational biomarker program	Test whether adding pembrolizumab improves OS/PFS over lenvatinib alone	[161]
NRG/RTOG 1112	III	Locally advanced HCC unsuitable for surgery	SBRT followed by sorafenib vs. sorafenib alone	Local therapy + systemic TKI; imaging and liver function biomarkers	Determine whether SBRT + sorafenib improves OS vs. sorafenib alone	[162]

### Impact of Proteogenomics in Melanoma Patient Outcomes and Precision Oncology

#### Notable Impact of KEYNOTE‑716 Trial on Patient Outcomes and Precision Oncology

The KEYNOTE‑716 trial evaluated adjuvant pembrolizumab versus placebo in patients with completely resected stage IIB or IIC melanoma, a population historically at high risk of recurrence despite surgery. The study has meaningfully shifted clinical practice in several ways [120].

1. Improved Recurrence‑Free Survival (RFS). The trial demonstrated a clear RFS benefit, establishing pembrolizumab as an effective adjuvant therapy for high‑risk stage II melanoma. This is a major step in precision oncology: identifying a molecularly targeted immunotherapy that benefits a specific, well‑defined risk group.
2. Improved Distant Metastasis‑Free Survival (DMFS). This is clinically meaningful because distant metastasis is the primary driver of melanoma‑related mortality. Preventing metastatic spread is one of the strongest indicators of long‑term survival benefit.
3. Refinement of Risk‑Stratified Care. By demonstrating benefit specifically in stage IIB/IIC melanoma, the study has refined risk stratification, helped clinicians identify which early‑stage patients benefit from systemic therapy and reduced reliance on “wait‑and‑see” approaches. This is precision oncology in action: matching treatment intensity to individualized recurrence risk.

#### Notable Impact of CheckMate 067 Trial on Patient Outcomes and Precision Oncology

1. Durable overall survival (OS) benefit. CheckMate 067 showed unprecedented long‑term survival in advanced melanoma, with a substantial proportion of patients alive at 5+ years on nivolumab–ipilimumab and durable benefit also with nivolumab monotherapy compared with ipilimumab alone. This established dual checkpoint blockade as a benchmark for long‑term disease control in metastatic melanoma [122].
2. Improved progression‑free survival (PFS) and depth of response. Combination therapy significantly improved PFS and objective response rates, including higher complete response rates, versus ipilimumab alone. These deep, durable responses became a clinical surrogate for long‑term benefit and informed how we think about “functional cure” in a subset of patients.
3. Long‑term health‑related quality of life (HRQoL). Despite higher acute toxicity with the combination, long‑term HRQoL in survivors on nivolumab±ipilimumab was generally maintained, supporting the idea that short‑term intensive toxicity can be acceptable when traded for durable disease control and prolonged survival.
4. Risk–benefit stratification rather than one‑size‑fits‑all. CheckMate 067 catalyzed a more nuanced, “precision” approach by the combination of nivolumab–ipilimumab for patients where maximal response probability justifies higher toxicity. Nivolumab monotherapy for patients where toxicity minimization is prioritized but a durable benefit is still likely.
5. Benchmark for future immunotherapy combinations. The trial set the standard comparator for new regimens (e.g., PD‑1 plus LAG‑3, TIGIT, or targeted agents). Any new strategy in advanced melanoma is now judged against the long‑term OS and PFS curves of CheckMate 067, effectively “raising the bar” for what counts as meaningful improvement.

### Impact of Proteogenomics in Hepatocellular Carcinoma Patient Outcomes and Precision Oncology

#### Notable Impact of KEYNOTE‑224 Trial on Patient Outcomes and Precision Oncology

1. The trial had a significant impact on a population that was previously difficult to treat. KEYNOTE‑224 was a phase II, single‑arm trial of pembrolizumab in patients with HCC previously treated with sorafenib. The study demonstrated clinically relevant objective response rates (~17%), with many responses durable and a substantial proportion of patients achieving disease control [154].
2. Durable responses and survival signal. Follow-up analyses showed that when patients responded, responses tended to be long-lasting, with a tail on the survival curve suggestive of long-term benefit.
3. Set the stage for line-of-therapy and combination questions. Subsequent analyses further explored how pembrolizumab as first-line monotherapy in advanced HCC within KEYNOTE-224 fits with PD-1 blockade, TKIs, and combinations, helping to define who might benefit from single-agent immunotherapy versus combination regimens.
4. Opened a new therapeutic avenue for patients progressing on sorafenib, where options were previously limited.

#### Notable Impact of COSMIC‑312 Trial on Patient Outcomes and Precision Oncology

1. The trial yielded incremental, rather than transformative, clinical benefits. COSMIC‑312 showed that adding cabozantinib to atezolizumab can slow down disease progression but does not clearly extend overall survival compared to sorafenib, unlike the survival improvements seen with other immune-oncology combinations [159].
2. The trial refined expectations for IO–TKI combinations. The trial tempered the early enthusiasm that the PD‑(L)1 + TKI pair would automatically deliver OS benefits.
3. Risk–benefit and cost‑effectiveness scrutiny.
4. COSMIC‑312 delivered a PFS benefit but not an OS benefit.COSMIC-312 serves as a comparator and control for future trials. COSMIC‑312 is now used as a standard treatment and data source for combining immunotherapy and targeted therapy in liver cancer, helping to clarify what benefits (like overall survival, quality of life, and side effects) are really important compared to existing treatments.

## Challenges in Proteogenomics

Despite the transformative potential of proteogenomics, its transition from a discovery tool to a routine clinical tool is hindered by significant technical, biological, and logistical challenges. These obstacles stem from the inherent complexity of the proteome compared to the genome and the difficulties associated with high-dimensional data integration. While integrative proteogenomic approaches have demonstrated substantial value in uncovering functional protein dependencies, resistance mechanisms, and clinically actionable signalling nodes, particularly in melanoma and other immunotherapy-responsive cancers, their broader translational impact remains constrained by several persistent challenges [29, 163, 164]. These limitations span pre-analytical variability, proteome coverage, computational integration, biological complexity, and clinical feasibility, collectively tempering the routine application of proteogenomics in precision oncology. Overcoming these barriers is essential to fully leverage proteogenomic insights for cancer diagnosis, stratification, and therapy selection [165, 166].

A fundamental challenge lies in sample acquisition and pre-analytical variability. Proteogenomic analyses require high-quality specimens suitable for parallel genomic, transcriptomic, and proteomic profiling, yet clinical tumor samples are often limited in quantity and heterogeneity [167, 168]. Factors such as cold ischemia time, fixation, and storage conditions can profoundly affect protein phosphorylation and other post-translational modifications (PTMs), even when global protein abundance remains largely unchanged [169, 170]. This sensitivity complicates the interpretation of signaling data and reduces the reliability of retrospective clinical cohorts unless stringent standard operating procedures are applied [165].

A primary hurdle in proteogenomics is the lack of biological amplification for proteins. Unlike DNA and RNA, which can be amplified via PCR or library synthesis, the detection of low-abundance proteins relies entirely on the sensitivity and dynamic range of mass spectrometry (MS) [171, 172]. In complex clinical samples, the dynamic range of protein concentration can exceed ten orders of magnitude, often leading to the ‘masking’ of low-copy-number signaling molecules by high-abundance structural proteins. Furthermore, proteogenomics requires high-quality, fresh-frozen tissue, as the phosphoproteome is extremely labile and susceptible to rapid degradation by endogenous phosphatases during warm ischemia [173]. This requirement often conflicts with standard clinical workflows that prioritize Formalin-Fixed Paraffin-Embedded (FFPE) preservation, which remains challenging for high-depth proteomic analysis.

Another major limitation is incomplete and biased proteome coverage. Unlike sequencing-based technologies, mass spectrometry-based proteomics does not yet achieve comprehensive detection of low-abundance proteins, membrane proteins, or condition-specific isoforms. Dynamic range constraints, peptide ionization inefficiencies, and stochastic sampling, particularly in data-dependent acquisition, result in missing values and limit the detection of variant peptides derived from somatic mutations or alternative splicing events [174, 175]. Consequently, many genomic alterations lack direct protein-level confirmation, weakening genotype-phenotype associations.

Additionally, computational and bioinformatics challenges further restrict scalability and reproducibility. Proteogenomic workflows generate large, multidimensional datasets that demand specialized computational infrastructure and expertise [176, 177]. The use of customized protein databases incorporating sample-specific variants substantially expands search space, increasing false discovery rates and complicating peptide identification [94]. Moreover, there is no universal standard for normalizing, integrating, or visualizing multi-omics data, which makes it difficult to compare studies and set clinical benchmarks [178].

Biological complexity presents an additional obstacle. Protein abundance and activity are shaped by translational regulation, degradation, subcellular localization, and PTMs, resulting in weak correlations between DNA, RNA, and protein levels. In cancer, this disconnect is amplified by intratumoral heterogeneity, clonal evolution, and tumor microenvironmental influences [179, 180]. Bulk proteogenomic analyses may obscure cell-type-specific signaling programs, while emerging single-cell proteogenomic approaches, although promising, remain technically challenging and resource-intensive [181].

Finally, clinical translation and cost-effectiveness remain significant barriers. Defined as the “time-cost-utility” triad [182]. Currently, the workflow for deep proteogenomics, encompassing sample preparation, multi-day MS runs, and integrated bioinformatic analysis, often exceeds this window. Moreover, the infrastructure cost of high-end equipment, e.g., mass spectrometers, and the need for specialized personnel limit this technology to Tier-1 academic centers [165, 183]. Proteogenomic analyses require advanced instrumentation, extended turnaround times, and highly specialized personnel, limiting their feasibility in routine oncology practice [184]. Regulatory validation of proteogenomic biomarkers and their integration into clinical decision-making frameworks are still in early stages, further delaying adoption [185, 186]. Only by overcoming these logistical and analytical hurdles can proteogenomics move beyond a descriptive tool to become the definitive “functional compass” for personalized cancer care.

In summary, while proteogenomics provides unprecedented functional insight into cancer biology, its clinical impact is currently constrained by technical, computational, biological, and translational challenges. Continued technological innovation, standardization, and prospective clinical validation will be essential to fully realize the promise of proteogenomic-driven precision oncology.

## Future Prospects of Proteogenomics in Precision Oncology

Proteogenomics is an evolving field whose progress is closely linked to advancements in related sub-disciplines. The future prospects can be analyzed through various interconnected perspectives, such as technological innovation, integrative data analysis, and the application of discoveries in clinical practice.

Recent advancements in single-cell and spatial genomics, transcriptomics, epigenomics, and proteomics are expected to enhance proteogenomic applications significantly, particularly in elucidating the complexities of the tumor microenvironment (TME) and improving therapeutic efficacy in cancer treatment [187]. Single-cell multi-omics methodologies in proteogenomics facilitate the development of tumor and treatment-response atlases, comprehensive characterization of molecular alterations, and the correlation of these modifications with clinical outcomes. Single-cell analyses are crucial for elucidating the mechanisms of therapeutic resistance in tumor and immune cells, clarifying treatment-related toxicities, and revealing dynamic changes in the tumor microenvironment before and during anticancer therapy. These approaches facilitate the identification of biomarkers for patient stratification, the discovery of novel cellular populations and protein effectors within the tumor microenvironment, and the assessment of organ-specific toxicities, thereby supporting more precise and personalized oncology strategies [187].

Data integration will enhance target discovery and validation in proteogenomic workflows by facilitating cross-validation of findings across genomic, transcriptomic, proteomic, and metabolic dimensions. Advancements in computational integration, particularly through statistical modeling and machine learning, are a critical focus for the next stage of proteogenomics. These methods facilitate a more comprehensive integration of genomic variation, transcript levels, and protein expression, enhancing the identification of regulatory mechanisms that underlie disease phenotypes. In oncology, integrative frameworks are anticipated to enhance connections among somatic mutations, dysregulated signalling networks, and therapeutic vulnerabilities, thus facilitating biomarker discovery and informed precision treatment design [94, 95].

Improvements in data visualization, standardization, and sharing is equally critical. With the increasing scale and complexity of proteogenomic datasets, the development of interoperable analytical pipelines, standardized data formats, and sophisticated visualization tools is crucial for ensuring reproducibility and enabling cross-study comparisons. Collaborative initiatives and community-driven resources serve as effective platforms for establishing best practices and enhancing access to high-quality public datasets, thereby accelerating methodological innovation and clinical translation [86]. Advancements in proteogenomics are anticipated to enhance its integration into clinical trials by providing a deeper mechanistic understanding across patient cohorts and identifying molecular factors that influence treatment response and resistance. Successful clinical implementation necessitates meticulous attention to sample acquisition, handling, and processing. The global proteome exhibits relative stability during standard handling times, whereas the phosphoproteome demonstrates significant dynamism, with biologically pertinent stress responses observable within minutes of cold ischaemia [188, 189]. Other post-translational modifications likely demonstrate comparable sensitivities, although they are not as well characterized. While pre-analytical variables in human studies cannot be eradicated, systematic and prospective collection, accompanied by thorough annotation, can reduce their influence. Although associated with increased costs and extended timelines, these approaches improve analytical reliability. Retrospective collections provide expedited access to longitudinal clinical data; however, they may not be appropriate for post-translational modification analyses unless gathered under stringent standard operating procedures [11].

## Conclusion

**Fig. 3:**
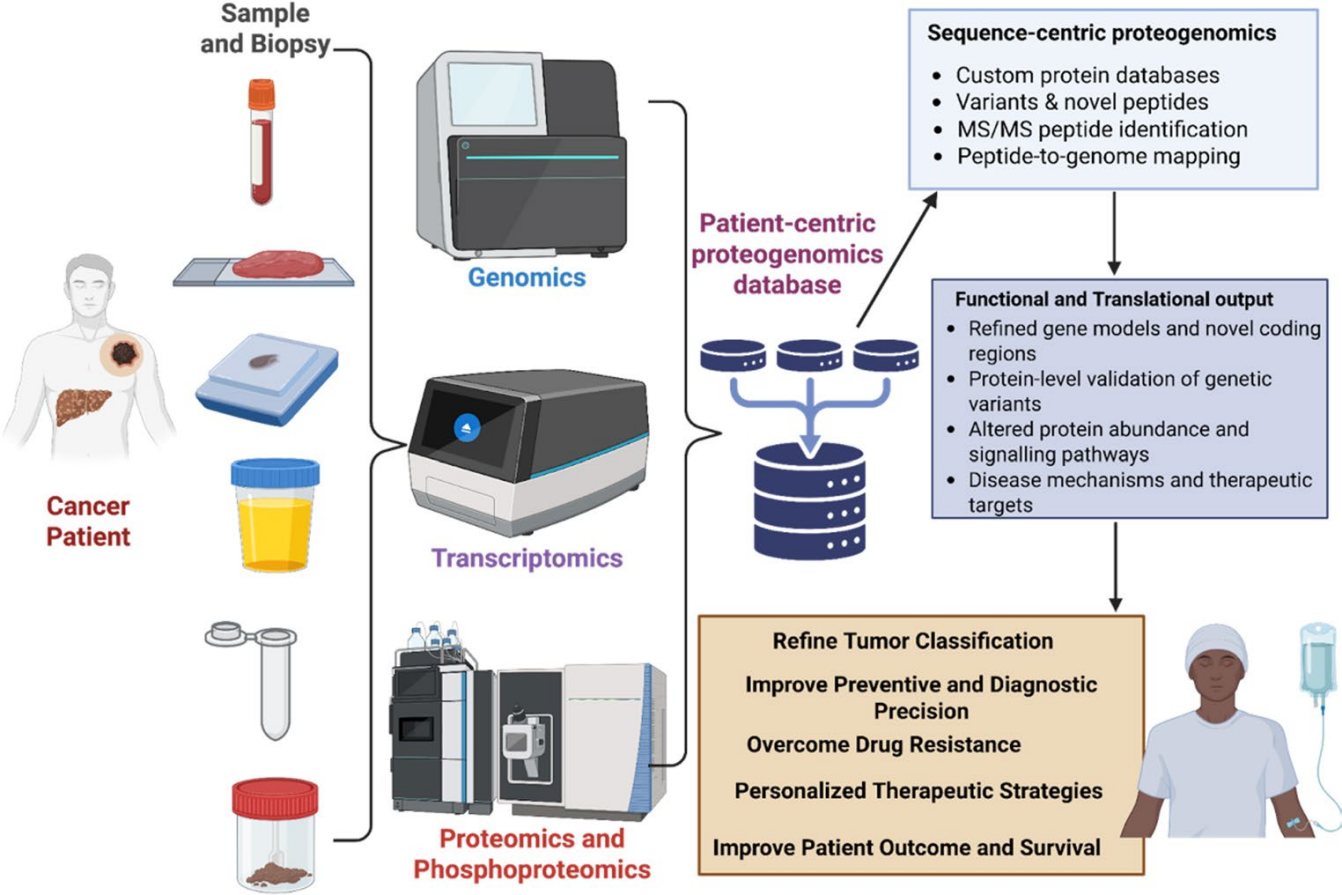
A schematic overview of the application of multi-omics technologies in precision oncology. This figure highlights the utilization of combined genomic, transcriptomic, proteomic, and phosphoproteomic data to characterize tumor heterogeneity, identify actionable molecular abnormalities, and inform personalized therapy regimens. The workflow emphasizes essential analytical stages, including sample gathering, multi-layer data collection, computational integration, biomarker identification, and clinical decision support. These integrated omics methodologies provide a thorough comprehension of tumor biology and aid in the formulation of accurate diagnostics, prognostic models, and targeted therapies in oncology (created on biorender.com)

Precision oncology has established itself as a cornerstone in the efficacy of present-day and future cancer care, as it improves cancer diagnosis, prognosis, treatment, and management, among other aspects. This preeminence is supported by high-throughput technologies such as genomics, transcriptomics, and proteomics, which reveal genetic and non-genetic signatures in tumors and patients – insights subsequently utilized to tailor therapeutic approaches. Despite their enormous contributions, the aforementioned tools are limited – genomics tools illuminate cancer-driving genetic alterations but are unable to delineate post-translational modification, protein amounts, and functions like proteomics techniques and this constrains the full potential realization of precision oncology. More integrative approaches such as proteogenomics are thus needed, especially for heterogeneous malignancies such as HCC and melanoma, to allow for comprehensive tumor profile analysis, tailored fit, and sustainable health interventions **(**Fig. 3**)**.

## Key References

- Mani DR, et al. Cancer proteogenomics: current impact and future prospects. *Nat Rev Cancer* 2022;22:298–313.
  - ○ This review establishes proteogenomics as a foundational framework for linking genomic alterations to functional protein networks, underpinning its central role in precision oncology across cancers, including melanoma and hepatocellular carcinoma.
- Joshi SK, et al. Mass Spectrometry-Based Proteogenomics: New Therapeutic Opportunities for Precision Medicine. *Annu Rev Pharmacol Toxicol* 2024;64:455–479.
  - ○ This review highlights mass spectrometry–based proteogenomics as a key approach for identifying actionable signaling pathways and resistance mechanisms beyond genome-centric analyses.
- Ng CKY, et al. Integrative proteogenomic characterization of hepatocellular carcinoma across etiologies and stages. *Nat Commun* 2022;13:2436.
  - ○ This study reveals proteogenomic signatures that stratify hepatocellular carcinoma by etiology and stage, uncovering functional networks not evident from genomic data alone.
- Xing X, et al. Integrated omics landscape of hepatocellular carcinoma suggests proteomic subtypes for precision therapy. *Cell Rep Med* 2023;4:101315.
  - ○ Integrated omics analysis identifies proteomic subtypes of hepatocellular carcinoma associated with therapeutic vulnerabilities and clinical outcomes.
- Long H, et al. Proteomic Characterization of Liver Cancer Cells Treated with Clinical Targeted Drugs for Hepatocellular Carcinoma. *Biomedicines* 2025;13:152.
  - ○ Proteomic profiling of drug-treated HCC cells reveals dynamic signaling responses that inform mechanisms of therapeutic sensitivity and resistance.
- Xiang H, et al. Proteogenomic insights into the biology and treatment of pan-melanoma. *Cell Discovery* 2024;10:78.
  - ○ Pan-melanoma proteogenomic analysis identifies conserved and subtype-specific signaling vulnerabilities that guide precision therapeutic strategies.
- Wahida A, et al. The coming decade in precision oncology: six riddles. *Nat Rev Cancer* 2023;23:43–54.
  - ○ This perspective frames key challenges in precision oncology and positions proteogenomics as a critical solution for addressing tumor heterogeneity, biomarker robustness, and clinical translation.
- Guedes J, et al. The melanoma MEGA-study: Integrating proteogenomics, digital pathology, and AI-analytics for precision oncology. *J Proteomics* 2025;319:105482.
  - ○ The MEGA study demonstrates that integrative proteogenomics, combined with digital pathology and AI, refines melanoma classification and therapeutic stratification in precision oncology.
- Piana D, et al. Phenotyping Tumor Heterogeneity through Proteogenomics: Study Models and Challenges. *IJMS* 2024;25:8830.
  - ○ This work shows how proteogenomic approaches resolve tumor heterogeneity and evolution, informing biomarker development and treatment response assessment.
- Safri F, et al. Heterogeneity of hepatocellular carcinoma: from mechanisms to clinical implications. *Cancer Gene Ther* 2024;31:1105–1112.
  - ○ This review underscores the clinical impact of molecular heterogeneity in hepatocellular carcinoma and supports the need for proteogenomic integration in patient stratification.
- Dawson LA, et al. Stereotactic Body Radiotherapy vs. Sorafenib Alone in Hepatocellular Carcinoma: The NRG Oncology/RTOG 1112 Phase 3 Randomized Clinical Trial. *JAMA Oncol* 2025;11:136–144.
  - ○ This phase 3 trial highlights the need for molecular and proteogenomic biomarkers to optimize patient selection for multimodal therapy in hepatocellular carcinoma.
- Weber JS, et al. Individualised neoantigen therapy mRNA-4157 (V940) plus pembrolizumab versus pembrolizumab monotherapy in resected melanoma (KEYNOTE-942). *Lancet* 2024;403:632–644.
  - ○ This trial demonstrates the clinical benefit of personalized neoantigen-based immunotherapy in melanoma, emphasizing the importance of integrated molecular profiling for individualized treatment.

## Data Availability

No datasets were generated or analysed during the current study.
